# SkinLesNet: Classification of Skin Lesions and Detection of Melanoma Cancer Using a Novel Multi-Layer Deep Convolutional Neural Network

**DOI:** 10.3390/cancers16010108

**Published:** 2023-12-24

**Authors:** Muhammad Azeem, Kaveh Kiani, Taha Mansouri, Nathan Topping

**Affiliations:** School of Science, Engineering & Environment, University of Salford, Manchester M5 4WT, UK; k.kiani@salford.ac.uk (K.K.); t.mansouri@salford.ac.uk (T.M.); n.j.topping@salford.ac.uk (N.T.)

**Keywords:** deep learning, convolutional neural network, computer vision, computer-aided diagnosis, skin lesion, skin cancer, melanoma, medical imaging

## Abstract

**Simple Summary:**

While melanoma accounts for 4% of skin cancer cases, it causes 75% of skin-cancer-related deaths. The survival rate for melanoma is higher for early-identified cases, so improved access to diagnosis and screening programs is essential for addressing skin cancer deaths. Computer-aided diagnosis utilizing machine learning can be used to differentiate malignant and benign skin lesions. There is significant research into the use of convolutional neural networks to classify skin lesions from dermoscopic images. However, to provide cost-effective and accessible options for early detection of malignant melanoma, smartphone applications capable of accurately classifying skin lesions from images taken on a smartphone would be beneficial. This research investigates a previously underexplored dataset of smartphone images and develops a novel multi-layer deep convolutional neural network model, named SkinLesNet, to classify three types of skin lesions, including melanoma. Further studies to validate the model should be conducted as other image datasets become available.

**Abstract:**

Skin cancer is a widespread disease that typically develops on the skin due to frequent exposure to sunlight. Although cancer can appear on any part of the human body, skin cancer accounts for a significant proportion of all new cancer diagnoses worldwide. There are substantial obstacles to the precise diagnosis and classification of skin lesions because of morphological variety and indistinguishable characteristics across skin malignancies. Recently, deep learning models have been used in the field of image-based skin-lesion diagnosis and have demonstrated diagnostic efficiency on par with that of dermatologists. To increase classification efficiency and accuracy for skin lesions, a cutting-edge multi-layer deep convolutional neural network termed SkinLesNet was built in this study. The dataset used in this study was extracted from the PAD-UFES-20 dataset and was augmented. The PAD-UFES-20-Modified dataset includes three common forms of skin lesions: seborrheic keratosis, nevus, and melanoma. To comprehensively assess SkinLesNet’s performance, its evaluation was expanded beyond the PAD-UFES-20-Modified dataset. Two additional datasets, HAM10000 and ISIC2017, were included, and SkinLesNet was compared to the widely used ResNet50 and VGG16 models. This broader evaluation confirmed SkinLesNet’s effectiveness, as it consistently outperformed both benchmarks across all datasets.

## 1. Introduction

“Cancer”—the collective term by which a group of linked diseases is referred to—occurs when several body cells start to divide uncontrollably and to invade nearby tissues [1]. Skin cancer is one of the most common forms of cancer [2] and commonly occurs when the skin is frequently exposed to sunlight [3]. Ultraviolet rays, which are the primary cause of skin cancer, harm the DNA in skin cells [4]. There are three main types of skin cancer: basal cell carcinoma, squamous cell carcinoma, and melanoma [5]. However, non-melanoma skin cancers present a lower risk of spreading to other parts of the body and are easier to treat than melanoma [6]. It is estimated that while melanoma only accounts for 4% of skin cancer cases, it causes 75% of skin-cancer-related deaths [7]. Globally, there were an estimated 287,700 new cases of melanoma in 2018 and an estimated 60,700 deaths from melanoma in the same year [2]. The incidence of melanoma has grown significantly in recent times, partly due to an increase in sun-seeking behaviors [7]. For example, in the United States, the lifetime risk of developing malignant melanoma has increased from 1 in 5000 in the year 1935 to 1 in 74 in the year 2000 [8].

Early-identified individuals have a better chance of recovering, because the five-year survival rate for patients with early-identified malignant melanoma is 94% [8]. Early diagnosis is therefore a critical factor in reducing skin cancer mortality. Dermoscopy is a specialist technology that produces high-resolution magnified images of the skin by controlling light and removing surface skin reflectance [9], and the clinical use of dermatoscopic images has the potential to improve diagnosis rates for melanoma [8] and, ultimately, to save lives [10]. However, given the existing pressures on healthcare systems, cost-effective strategies are needed to facilitate increased screening and diagnosis [11], and there has been significant interest in computer-aided diagnosis [6]. An active area of research is the use of smartphone applications incorporating machine-learning methods to analyze images and assist in early melanoma diagnosis, whilst reducing pressures on healthcare systems and clinical staff [12].

Clinicians have traditionally utilized the ABCD guidelines to differentiate melanoma from non-malignant skin lesions, with A denoting asymmetry, B denoting border irregularity, C denoting color variations, and D denoting diameter greater than 6 mm [8,13]. However, due to the morphological variation and complex characteristics of skin lesions, there can be challenges with inter-observer and intra-observer concordance, further motivating the exploration of computer-aided diagnosis techniques [14]. By utilizing texture cues, geometrical aspects, color features, and combinations of these features, medical images can be used to identify and classify skin cancer conditions [15]. The use of traditional machine-learning techniques in melanoma diagnosis has typically involved feature extraction from dermatoscopic images, to build a set of relevant features that can be used to train a classification model, often utilizing the ABCD rule to define an appropriate feature set [14,16,17,18]. Due to the complexity of skin lesions, it is difficult for researchers to recognize skin malignancies by using these geometrical properties [18].

In the field of image-based melanoma diagnosis, the development of deep learning and, in particular, convolutional neural networks (CNNs) has reduced reliance on manual-feature extraction techniques. CNN-based classification methods have also demonstrated diagnostic effectiveness comparable to that of dermatologists [19]. In [20], researchers mainly concentrated on the automatic identification and categorization of skin cancer, as computer-aided screening technologies had become more prevalent. Many studies have been conducted to categorize melanoma skin lesions, using CNNs on various datasets, including MNIST HAM10000 [21], the International Skin Imaging Collaboration 2018 (ISIC) [22], the PH2 public database [23], and the International Symposium on Biomedical Imaging (ISBI) [24]. Using these datasets, promising results have been achieved, employing a variety of pre-trained CNN models, including ResNet50 [25], ImageNet50 [26], and DenseNet201 [27] alongside additional cutting-edge models. Some datasets, such as the Dermatological and Surgical Assistance Program at the Federal University of Espirito Santo (PAD-UFES-20) dataset [28], have not been explored as extensively for the identification of skin lesions.

In order to classify and discriminate skin lesions, we have created and used a modified version of the PAD-UFES-20 dataset, named PAD-UFES-20-Modified. The PAD-UFES-20-Modified dataset was chosen for this research due to its unique characteristics and practicality. This dataset consists of 1314 samples that correspond to three primary types of skin lesions: nevus, melanoma, and seborrheic keratosis. The dataset was collated from images taken using smartphones, meaning it is particularly well suited to investigating the possibility of melanoma detection and diagnosis through smartphone applications [28]. The integration of patient medical data offers insightful contextual information for each lesion. The model’s ability to undergo thorough training and assessment is strengthened by the contextual richness, the number of samples in the dataset, and the thorough annotations. This dataset’s clinically significant features were in alignment with the objectives of this study. The choice of PAD-UFES-20-Modified, with its diverse samples and comprehensive information, aligns with the complexity of real-world scenarios, making it a valuable resource for developing and testing the SkinLesNet model.

### Contributions

The introduction provides an overview of the challenges posed by skin cancer, the importance of early detection, and the prevalence and severity of melanoma. The following points highlight the key contributions of the paper:

- The development and implementation of a cutting-edge multi-layer CNN model represents a significant contribution. The model was specifically designed for the classification and discrimination of skin lesions, and its superior performance—achieving a 96% accuracy rate—demonstrates its effectiveness, compared to established models like ResNet50 and VGG16.
- This research contributes to the field by utilizing the PAD-UFES-20 dataset, which has not been as extensively explored for skin-lesion classification. This dataset contains smartphone images rather than dermatoscopic images, which is particularly relevant to the development of smartphone applications for accessible, scalable, and cost-effective melanoma diagnosis.
- This study evaluated the proposed model on diverse datasets, including the PAD-UFES-20-Modified dataset, HAM10000, and ISIC2017. This approach enhanced the generalizability of the model, showcasing its adaptability to different datasets and real-world scenarios.
- This study’s primary contribution lies in achieving a high accuracy rate of 96% in classifying skin lesions. This is a crucial contribution, considering the complexities and challenges associated with accurate dermatological diagnoses.

The paper has been structured as follows: Section 2 briefly highlights the related work; Section 3 explains the methodology used to clean and preprocess the dataset, and to build and train the model; Section 4 discusses the results and evaluation metrics of the models; Section 5 contains the conclusion.

## 2. Literature Review

Machine learning and AI have made significant advances in cancer prediction and detection in recent years [29]. Dermoscopy is a non-invasive imaging method for taking comprehensive images of skin lesions [30]. The development of computer-aided diagnosis systems has been spurred by research into the need for accurate and early identification of skin illnesses, including melanoma and other types of skin cancer [31]. When used to automate the classification of skin lesions based on dermoscopy images, deep learning methods, in particular CNNs, have demonstrated encouraging results [32]. Earlier methods for classifying skin lesions relied on manually engineered characteristics and conventional machine-learning techniques [33]. The use of deep learning techniques, particularly CNNs, has reduced the reliance on manual feature extraction in skin-lesion classification [34]. For classification tasks involving skin lesions, well-known CNN architectures like AlexNet [35], VGGNet [36], and InceptionNet [37] have been adapted and refined.

Deep learning models were developed by [38], where CNN models were trained and evaluated on the HAM10000 dataset, which delivered 90 per cent validation accuracy when classifying various forms of skin malignancies. In [39], with the help of a data augmentation technique, a CNN classification model was proposed and trained, using a public dataset of skin lesions that included 600 test and 6162 training images, achieving a classification accuracy of 89.2%. Using a cutting-edge prediction algorithm, benign and malignant skin lesions were separated into categories in [40] with a CNN and a novel regularizer. The model was then trained with a dataset obtained by the International Skin Imaging Collaboration (ISIC) databank, which acquired a summed accuracy score of 97.49%. In [41], by using fuzzy C-means clustering and K-means clustering, the researchers classified skin lesions using a CNN model trained with the ISIC dataset, achieving an accuracy of 98.83%.

In [42], transfer-learning techniques were used with two CNN architectures, ResNet50 and DenseNet169. The models were trained and validated on the HAM10000 dataset, and the highest-performing generated an accuracy score of 91.2%. In addition to existing methods, such as border extraction utilizing XOR with regression logic, another CNN model was suggested in [43]. The datasets from PH2 and ISBI 2017 were utilized to train the model, which achieved a 97.8% accuracy rate. In [44], to enhance the performance of the proposed CNN model, a transfer-learning strategy was employed, using a publicly available dataset on Kaggle, which resulted in accuracy of 79.45%. Another CNN model was designed by [45] and trained with a medical dataset acquired from Al-Kindi Hospital and Baghdad Medical City to classify skin lesions, obtaining accuracy of 89%. In [46], seven different types of skin problems were categorized, using a CNN.

In [47], a U-Net-based model was proposed for semantic segmentation of skin-lesion images, and the proposed model was validated against ISIC2018, ISIC2017, and PH2. In [48], a U-Net model was also used for skin lesion semantic segmentation and was evaluated with ISIC2017 and ISIC2018, with accuracy of 94.9% and 95.4%, respectively. In [49], a CNN model that distinguished blemishes into moderate skin cancer and cases of acne was developed, using images of diverse benign skin cancers and acne cases, and it yielded precision of 96.4%. In [50], a dataset of skin cancer dermoscopy images was utilized, subjected to a number of data-cleaning steps to reduce noise and enhance the quality of images, and then a CNN model was employed for categorization, achieving accuracy of 98.38%. In [51], the HAM10000 dataset was used to train a Siamese neural network. While the classification accuracy was lower than with some other models, this approach was able to detect examples that did not belong to the training classes.

In [52], the PH2 dataset of dermoscopic images was utilized to create and build a CNN model, which achieved test-set accuracy of over 95%. In [53], a six-layer CNN model was created and trained on the ISIC dataset and showed promise, with accuracy of 89.30% in classifying skin lesions. Another State-of-the-Art CNN model was designed and developed by [54]. The model achieved 97.50% accuracy results when used with the ISIC and PH2 datasets, to separate skin lesions.

In [55], along with data augmentation and image preparation procedures, a CNN model, which obtained 95.2% accuracy, was trained and tested on the HAM10000 dataset. In [56], a CNN model was designed and trained on the ISIC2019 dataset, successfully classifying eight types of skin malignancies with a 94.92% test-accuracy score. In [57], the DenseNet201 model was fine-tuned and trained on the HAM10000 dataset, to classify skin lesions in dermoscopy images, obtaining 86.91% test accuracy. In [58], a deep CNN was created and trained on the ISBI 2017 dataset, to classify melanoma skin lesions. This network achieved 87% accuracy on test data.

Table 1 presents detailed performance metrics and a comparative analysis of the implemented CNN models on each dermoscopy dataset.

## 3. Methodology

To develop the SkinLesNet model, the Keras and TensorFlow Python libraries were used. Google Colab, which is a cloud-based platform, was used to execute the SkinLesNet model’s Python code. Google Colab was built on top of the Jupyter Notebook infrastructure.

The performance of the SkinLesNet model was compared to the ResNet50 and VGG16 models. ResNet50 [60] and VGG16 [61] were selected as benchmarks because they are popular CNN architectures known for their effectiveness in image-classification tasks, including medical-imaging applications [62,63,64,65]. ResNet50, part of the ResNet family, utilizes skip connections that aid in mitigating the vanishing gradient problem during training, enabling the network to effectively learn from a broader set of features [62]. On the other hand, VGG16 is recognized for its simple and uniform architecture with multiple convolutional layers, which makes it efficient in learning various image representations [63]. Both models have demonstrated strong performance in image-based tasks, due to their ability to extract meaningful features from medical images, thus making them suitable choices for skin cancer detection.

### 3.1. Dataset and Data Augmentation

A voluntary program at the Federal University of Espirito Santo (UFES), known as The Programa de Assistencia Dermatologica e Cirurgica (PAD), contributes handout skin-lesion medication, mostly to deserving individuals who are unable to pay for personal medical services. The 19th century witnessed millions of immigrants from Europe settle in the Espirito Santo state, for reasons of historical significance. The majority of those immigrants and their children were unprepared for Brazil’s tropical atmosphere. Because skin cancer and skin lesions are so common in this state, PAD is crucial in helping those who are affected. Because they were taken with different devices, the images in this collection have different resolutions, sizes, and lighting. To accurately detect skin cancer, this heterogeneity must be addressed.

The initial PAD-UFES-20 dataset exhibited a composition of 52 melanoma, 244 nevus, and 235 seborrheic keratosis images, as well as several other categories of benign and malignant skin lesions. Due to the risks associated with melanoma [7], this was the focus of the study. Misdiagnosis of melanoma as seborrheic keratosis or nevus could lead to suboptimal patient outcomes, yet these are two of the most common misdiagnoses [66,67].

The PAD-UFES-20-Modified dataset, comprising 1314 samples of seborrheic keratosis, nevus, and melanoma, stands out for its diversity and real-world relevance, as it includes both clinical images and patient medical records. During the data preprocessing stage, the images are standardized to 224 × 224 pixels and are split into training and test sets, to ensure model generalizability. The choice of PAD-UFES-20-Modified, with its diverse samples of smartphone images and comprehensive information, aligns with the complexity of real-world scenarios, making it a valuable resource for development and testing the SkinLesNet model.

**Figure 1:**
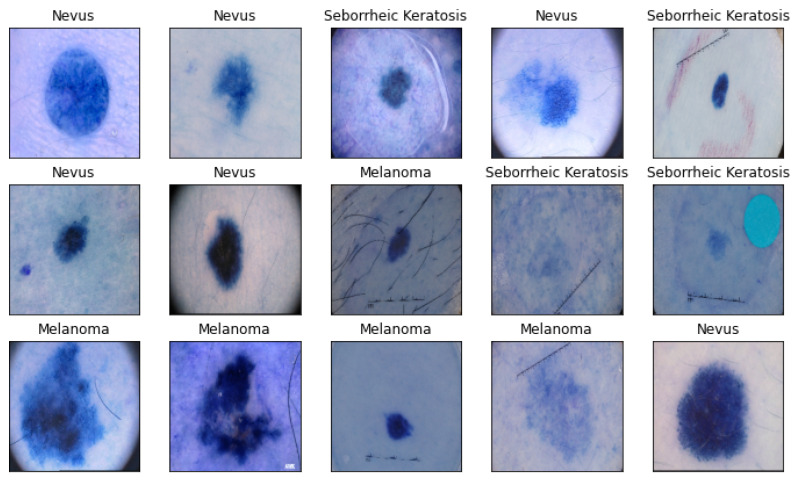
Illustrative representations from the PAD-UFES-20-Modified dataset employed in this research exhibit diverse visualizations of distinct skin lesions, encompassing the three respective categories of seborrheic keratosis, nevus, and melanoma.

In the utilization of the PAD-UFES-20-Modified dataset, a data augmentation strategy was employed, to address the challenges posed by imbalanced class distributions and limited original data. To enhance the dataset’s diversity and to mitigate the risk of model bias, a geometric-transformation [68] data-augmentation technique was implemented, introducing variations through random flips and rotations or translations. Consequently, the dataset was substantially expanded, yielding 520 melanoma, 408 nevus, and 386 seborrheic keratosis images, totalling 1314 images. Figure 1 provides examples from the image collections utilized in this investigation.

**Figure 2:**
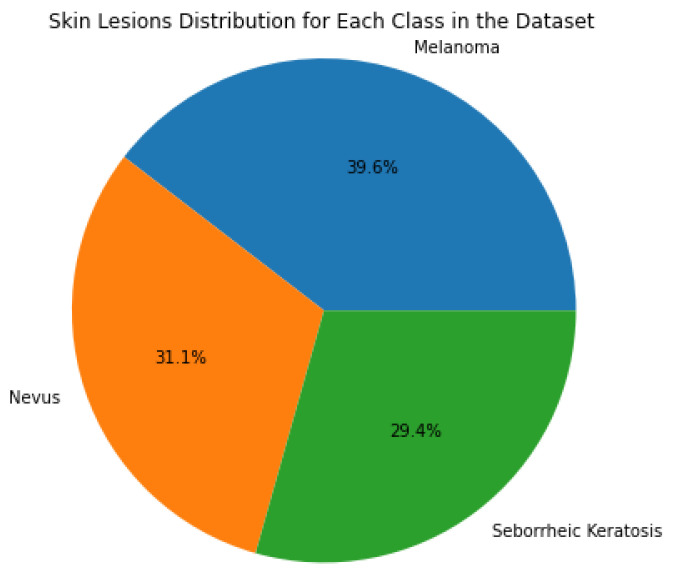
The pie chart highlights the distribution of different skin-lesion classes within the PAD-UFES-20-Modified dataset, and shows that in this dataset there is no significant class imbalance.

The distribution of the three skin lesion classes is shown in a pie chart in Figure 2. The pie chart effectively visualizes class balance or imbalance within the dataset, making it easier to grasp the relative proportions of the three classes.

To achieve uniformity and compatibility with the deep learning model’s input size requirements, it was necessary to standardize the image size. Every image was therefore reduced in size to a square with dimensions of 224 × 224 pixels.

The dataset was split into training and test sets in an 80:20 ratio, with 80% of the data being used for training and 20% being held back for testing, Table 2. To ensure that classes were distributed randomly across the sets used for training and testing, the train_test_split function randomly shuffled the data before splitting. This division was compulsory, to assess the model’s generalizability to new data during testing and to guard against overfitting.

### 3.2. Comparative Datasets

Apart from our primary dataset for this research, we used two more well-known and publicly available datasets, HAM10000 [69] and ISIC2017 [70]. The HAM10000 dataset, also known as “Human Against Machine with 10,000 training images”, is a set of dermatoscopic images of skin lesions. It comprises 10,015 dermatoscopic skin lesion images. The skin lesions in the dataset are divided into many categories, such as basal-cell carcinoma, squamous-cell carcinoma, seborrheic keratosis, melanoma, and nevus (moles) [71]. As melanoma is the most deadly type of skin cancer, it is of special interest. Furthermore, the International Skin Imaging Collaboration (ISIC), a global initiative to advance the early detection and diagnosis of skin cancer, particularly melanoma, includes the ISIC2017 dataset. A sizable number of dermatoscopic images of skin lesions are included in the dataset. The dataset’s skin lesions are divided into a number of classifications, with a particular emphasis on melanoma, the deadliest type of skin cancer. Basal-cell cancer, seborrheic keratosis, and nevus (moles) are possible further classes [72].

### 3.3. Proposed Model Architecture

**Figure 3:**
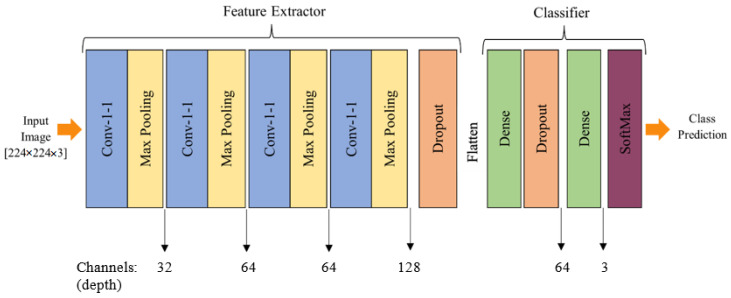
Proposed multi-layer deep CNN model architecture to classify different skin lesions categories.

We thoroughly tested numerous layer combinations in our neural network architecture before recommending a four-layer CNN model. The number of convolutional layers, the kind and size of filters, the activation functions, and the presence of pooling layers were all adjusted during these analyses. Finding the best architecture that balanced model complexity and efficiency was our goal. To make sure the model could correctly diagnose skin lesions, we evaluated several configurations, using measures including accuracy, precision, recall, and F1-score. After extensive testing and analysis, we came to the conclusion that the four-layer CNN architecture, dubbed SkinLesNet, had the most promising outcomes, in terms of precision and robustness, supporting its recommendation as the final model for skin-lesion classification, Figure 3.

The decision to utilize a multi-layer CNN was grounded in its proven efficacy in learning hierarchical features from complex image data, particularly in the domain of medical image analysis. Studies in image-based melanoma diagnosis have consistently shown that deep CNNs can achieve diagnostic effectiveness comparable to dermatologists. The unique strength of the SkinLesNet model lies in its four-layer architecture, systematically optimized through extensive testing. The successive convolutional layers act as feature extractors, capturing intricate patterns in skin-lesion images. The inclusion of max-pooling layers aids in spatial-dimension reduction while retaining essential features. The ReLU activation function adds non-linearity to the model [73], allowing it to recognize complex patterns in the data. This architecture is adept at processing diverse features, including texture cues, geometrical aspects, and color features, crucial for accurate skin-lesion classification. The choice of this model, with its distinct architecture and robust performance metrics, positions SkinLesNet as a unique and effective approach compared to other methods, providing a strong foundation for feature extraction and contributing to superior predictions and classifications in the realm of skin-lesion diagnosis.

The input layer accepted RGB images with a resolution of 224 × 224. The ReLU activation function was used to capture image characteristics in the first convolutional layer, which included 32 filters with a 3 × 3 filter size. It was followed by a max-pooling layer that shrank the spatial dimensions. The model comprised three additional convolutional layers, each with additional filters. Each convolutional layer was followed by a further max-pooling layer. To avoid overfitting, a dropout layer with a rate of 0.5 was added. The ReLU activation function was then used to flatten the data before a final fully connected hidden layer with 64 neurons.

An additional dropout layer with a dropout rate of 0.3 preceded the output layer, which consisted of three output neurons utilizing a SoftMax activation function, to calculate probabilities for each class. The model was trained to produce precise estimations and to decrease classification errors, using previously unseen images of skin lesions. The Adam optimizer was chosen, with a learning rate of 0.001 and a moving average with an exponential momentum of 0.99, in order to improve the efficacy of training. As an integer representation of the target labels, “sparse_categorical_crossentropy” was used as the loss function in the model. The model’s performance was assessed using the accuracy statistic, which provided the percentage of labels that were correctly predicted during training and evaluation.

The CNN model was built up to be used for training on the skin lesions dataset with these options. As part of its training process, the CNN model analyzed the data, using the Adam optimizer to adjust its internal parameters and assess how well it anticipated the development of new skin lesions.

### 3.4. Model Training

Various metrics, including training and validation accuracy, loss, and other pertinent metrics, were used to track progress throughout the training phase. These metrics offered information about the model’s performance and aided in choosing how to adjust its hyperparameters and architectural design. The training phase, in general, was a data-driven, repeating procedure where the model worked to determine significant trends and abstractions from the data used for training, in order to produce accurate predictions on new, unseen data. A batch size of 32, 100 training epochs, and a validation split of 0.2 were used during training. These hyperparameters are summarized in Table 3.

The model frequently converged effectively during training at a moderate learning rate, such as 0.001. With smaller learning rates, overshooting or divergence—which may have happened—were avoided by allowing the model to make incremental weight adjustments. A lower learning rate made the optimization process more precise and controllable, which helped the model become broader and less prone to overfitting [74]. Smaller batch sizes bring noise into the gradient estimates, which can function as a type of regularization and aid in avoiding overfitting. Working with less data can be advantageous when this regularization effect is present. Batch sizes of up to 32 are memory-efficient and allow deep neural networks to be trained even on computers with little GPU capacity [75].

Convergence could be accelerated by the use of Adam optimization [76] of the learning rates during training. For each parameter, it maintained two moving averages: the first moment’s mean and the second moment’s uncentered variance. The optimizer could modify the learning rates, based on how the gradient behaved for each parameter using these moving averages. Adam is suitable for a variety of deep learning problems and is robust to noisy gradients.

## 4. Results and Discussions

The model’s performance on unseen data after it had been trained is displayed methodically. Performance metrics, such as accuracy, precision, recall, and F1-score, could help to determine the model’s strengths and flaws. The discussion of visuals, confusion matrices, and comparison to testing methods all helped to give a greater understanding of the model’s possibilities and limitations. Overall, the findings and analysis provide both practitioners and scholars in the field of medical image analysis with meaningful data that bridge the gap between the theoretical model’s conceptualization and its practical implementation.

**Figure 4:**
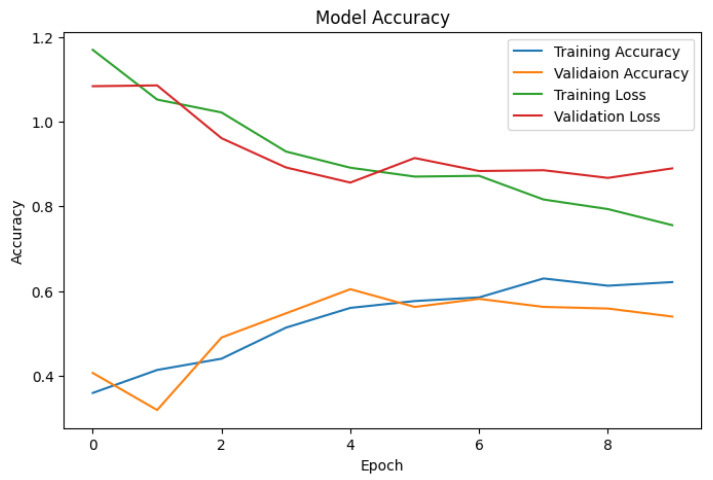
The graph depicts variations in training and validation accuracy and loss of the proposed SkinLesNet model over the first 10 epochs. Accuracy gradually increased and reached 96% after 100 epochs.

Figure 4 provides a detailed view of SkinLesNet’s early training stages, showcasing the evolution of both accuracy and loss metrics over the first 10 epochs. Observe the gradual ascent of training and validation accuracy, reaching a promising 96% after approximately 100 epochs. However, a closer examination of the loss curves within the figure offers valuable clues about the model’s optimization process and potential for further improvement.

ResNet50 contains several convolutional-layer combinations with average-pooling layers and batch-normalization layers. The fully connected layer, which has 1000 out-features, is the last layer in the original ResNet50 model. This work involved replacing this fully connected layer with a collection of fully connected layers, in order to fine-tune the ResNet50 model. There were 2048 out-features in the first fully connected layer. A probability of 0.5 was then applied in a dropout layer. The first and second fully connected layers were identical and used a ReLU activation function. Dropout with a probability of 0.5 was performed again after the second fully connected layer. There were three out-features and 2048 in-features in the final fully connected layer, which was intended for three-class categorization. In the VGG16 model, some layers were frozen and unfrozen, in order to fine-tune the model. Meanwhile, the VGG16 model was fine-tuned by unfreezing the last block, so that their weights were updated during training.

The proposed SkinLesNet model was first trained and tested on the PAD-UFES-20-Modified dataset, where it achieved 96% testing accuracy, with precision, recall, and F1-scores of 97%, 92%, and 92%, respectively. Moreover, the testing accuracies obtained for the ResNet50 and VGG16 models were 82% and 79%, Table 4.

The SkinLesNet model was then trained and tested on the HAM10000 dataset, where it achieved 90% testing accuracy, with precision, recall, and F1-scores of 89%, 87%, and 85%. Moreover, the testing accuracies obtained for the ResNet50 and VGG16 models were 80% and 75%, Table 5.

Finally, the SkinLesNet model was trained and tested on the ISIC2017 dataset, where it achieved 92% testing accuracy, with precision, recall, and F1-scores of 80%, 82%, and 75%, respectively. Moreover, the testing accuracies obtained for the ResNet50 and VGG16 models were 75% and 70%, Table 6.

The SkinLesNet model significantly outperformed all three datasets as compared to the ResNet50 and VGG16 models. The variations in accuracy across the different datasets can be attributed to the inherent differences in the dataset characteristics, complexities, and levels of diversity. While the PAD-UFES-20-Modified dataset achieved a notable accuracy of 96%, outperforming other datasets, such as HAM10000 and ISIC2017, several factors contributed to these differences. The PAD-UFES-20-Modified dataset, with its focus on clinical relevance and diverse lesion representation, aligns closely to the target application, fostering robust model performance. On the other hand, the HAM10000 and ISIC2017 datasets, although widely used, may exhibit variations in lesion types, distributions, or contextual information, potentially posing challenges for accurate classification. Variations in dataset sizes and annotation quality can influence model learning.

Despite these promising results, it is essential to acknowledge potential limitations and challenges in the comparison. The choice of datasets, while diverse, may not have encompassed the full spectrum of skin-lesion variations encountered in real-world clinical settings. The model’s performance may also have been influenced by the quality and quantity of data available for training. As with any deep learning model, overfitting remains a concern, although dropout layers were incorporated to mitigate this issue. Continuous efforts in data augmentation and the inclusion of larger and more diverse datasets could further enhance the model’s generalizability. Furthermore, the computational resources required for training and evaluating these models should be considered, especially as the complexity of the architecture increases. Despite these challenges, the SkinLesNet model’s consistently superior performance suggests its potential for practical implementation in dermatological applications, pending further refinement and validation.

## 5. Conclusions

SkinLesNet’s performance, which was discussed in the results section, demonstrated instances of accuracy and suggested areas for improvement. Two benchmark CNN architectures—VGG16 and ResNet50—were analyzed and compared to the proposed SkinLesNet model in this study. The PAD-UFES-20-Modified dataset was used to train and test the SkinLesNet model, which provided accuracy of 96%, compared to 82% for the ResNet50 model and 79% for the VGG16 model. Moreover, two other publicly available datasets were used to train the model: HAM10000 and ISIC2017. The model acquired accuracy on the HAM10000 and ISIC2017 datasets of 90% and 92%, respectively. Therefore, the SkinLesNet model outperformed the two benchmark models trained and tested on all three datasets. With sufficient computational resources and a well-annotated dataset, significant enhancements in model performance are achievable. Expanding the dataset and employing techniques like active learning or self-supervised learning could further improve model performance. Furthermore, while the problem is currently addressed through image classification, exploring the utilization of semantic-segmentation models could offer another effective approach to tackling this problem.

## Figures and Tables

**Table 1:** Comparison of implemented CNN models for skin lesions detection on different dermoscopy datasets.

Ref.	Model	Dataset	Accuracy	Comments
[38]	Deep convolutional neural network	HAM10000	90%	One of the main benchmark datasets was used in this paper, which produced promising results while using a CNN model. However, hyperparameters tuning is required, to increase the accuracy results.
[59]	Convolutional neural network (CNN)	International Skin Imaging Collaboration (ISIC)	97.49%	A CNN model was used, which showed relatively good results, but the size of the dataset needs to be maximized.
[42]	ResNet50	MNIST: HAM10000	91%	A State-of-the-Art model was used, which produced reasonable results on the given dataset. However, the dataset needs to be preprocessed well before training, to obtain more accurate and promising results.
[43]	Deep convolutional neural network	International Symposium on Biomedical Imaging (ISBI)	97.8%	A CNN model was trained on an internationally recognized benchmark dataset. However, the size of the dataset was decreased, which showed good results but could lead to model overfitting.
[48]	U-Net	International Skin Imaging Collaboration (ISIC)	94.9%	A State-of-the-Art model was used, which showed promising results, but more data preprocessing or augmentation is needed for accurate prediction.

**Table 2:** Number of images per class and train-test dataset split.

Dataset	Train (80%)	Test (20%)	Total
Melanoma	416	104	520
Nevus	326	82	408
Seborrheic			
Keratosis	309	77	386
**Total**	1051	263	1314

**Table 3:** Hyperparameters and configurations used to train the proposed multi-layer model for this work.

Learning Rate	Batch Size	Epochs	Optimizer	Activation
0.001	32	100	Adam	ReLU

**Table 4:** Performance comparison of SkinLesNet to other State-of-the-Art fine-tuned models for PAD-UFES-20-Modified test dataset.

Performance Metrics	VGG16	ResNet50	SkinLesNet
Accuracy	79%	82%	96%
Precision	80%	85%	97%
Recall	75%	75%	92%
F1-Score	72%	75%	92%

**Table 5:** Performance comparison of SkinLesNet to other State-of-the-Art fine-tuned models for HAM10000 test dataset.

Performance Metrics	VGG16	ResNet50	SkinLesNet
Accuracy	75%	80%	90%
Precision	75%	80%	89%
Recall	70%	72%	87%
F1-Score	70%	71%	85%

**Table 6:** Performance comparison of SkinLesNet to other State-of-the-Art fine-tuned models for ISIC2017 test dataset.

Performance Metrics	VGG16	ResNet50	SkinLesNet
Accuracy	70%	75%	92%
Precision	70%	75%	80%
Recall	70%	65%	82%
F1-Score	72%	70%	75%

## Data Availability

The data presented in this study are available on request from the corresponding author.

## GitHub Repository

The proposed SkinLesNet model is publicly available and accessible on the GitHub repository at the following link: SkinLesNet_Project.
